# Significance of a 4-week home-based prehabilitation program in accelerating 3-month recovery post total knee arthroplasty: a retrospective cohort study

**DOI:** 10.1186/s43019-026-00315-7

**Published:** 2026-07-01

**Authors:** Haiqi Ding, Guoyu Yu, Long Chen, Xiang Luo, Guiguan Wang, Hanhao Dai, Chen Chen, Jun Luo, Yuan Lin, Jie Xu, Fenqi Luo

**Affiliations:** 1 School of Medicine, Fuzhou University Affiliated Provincial Hospital, Fuzhou University, Fuzhou, Fujian China; 2 Department of Orthopedic, Fujian Provincial Hospital, Fuzhou, Fujian China; 3 Fujian Provincial Clinical Medical Research Center for Spinal Nerve and Joint Diseases, Fuzhou, Fujian China; 4 Chinese Academy of Medical Sciences and Peking Union Medical College, Beijing, China

**Keywords:** Preoperative rehabilitation, Osteoarthritis, Total knee arthroplasty, Enhanced recovery after surgery, Functional recovery

## Abstract

**Background:**

Preoperative rehabilitation is one of the strategies for enhanced recovery after surgery (ERAS) following total knee arthroplasty (TKA), but the optimal duration remains inconclusive. This study aims to evaluate the impact of a 4-week home-based prehabilitation program on accelerating postoperative recovery in patients undergoing TKA.

**Methods:**

In this retrospective cohort analysis, 176 patients undergoing primary unilateral TKA were categorized into two groups: those who completed a 4-week home-based prehabilitation program (training group, *n* = 72) and those who did not (control group, *n* = 104). Baseline demographics, perioperative data, pain scores, and functional outcomes were collected. Patients were followed for over 1 year. The primary outcome was the Western Ontario and McMaster Universities Arthritis Index (WOMAC), and secondary outcomes included visual analog scale (VAS), knee range of motion (ROM), Knee Society Score (KSS), timed up-and-go (TUG) test, and stair climbing test. Assessments were performed at baseline, before surgery, and multiple time points after TKA.

**Results:**

Baseline characteristics were comparable between groups. The training group demonstrated significantly earlier first postoperative ambulation (median 9 versus 12 h, *P* = 0.003) and shorter hospital stays (median 7 versus 10 days, *P* = 0.002). Pain scores (VAS) were significantly lower in the training group at 1 day and 1 week postoperatively (*P* < 0.05). Functional outcomes including ROM, KSS, TUG, and stair test were superior in the training group at 1 and 3 months (*P* < 0.05). WOMAC total scores and its subscales (pain, stiffness, function) also showed significant improvements in the Training group at 1 and 3 months (*P* < 0.05). While advantages in ROM and TUG persisted up to 6 months, no significant between-group differences were observed at 12 months for any outcome measure.

**Conclusions:**

A 4-week home-based prehabilitation program significantly enhances early recovery after TKA, as evidenced by reduced hospital stay, lower early postoperative pain, and improved functional outcomes within the first 3 months. Although benefits in certain functional measures persist up to 6 months, outcomes converge by 12 months. These findings support the integration of structured 4-week home-based prehabilitation program into ERAS pathways.

**Supplementary Information:**

The online version contains supplementary material available at 10.1186/s43019-026-00315-7.

## Introduction

Knee osteoarthritis (OA) is the leading cause of chronic pain and disability in adults over 50 years, and total knee arthroplasty (TKA) remains the definitive surgical option when conservative treatments fail [1, 2]. Despite advances in prosthetic design and perioperative care, a significant proportion of patients fail to achieve clinically meaningful improvements in early postoperative quality of life [3–5].

This clinical reality has driven a paradigm shift from a purely surgical–technical focus toward patient-centered, whole-course optimization strategies, recognizing that recovery optimization must begin preoperatively. Prehabilitation has emerged as an integral component of the enhanced recovery after surgery (ERAS) philosophy, on the basis of its capacity to augment functional muscle reserve, enhance neuromuscular coordination, and optimize joint proprioception [6, 7]. Research over the past decade has been characterized by substantial heterogeneity in the duration, content, and outcome measures of prehabilitation programs, precluding a definitive consensus on the efficacy of prehabilitation in accelerating recovery following total knee arthroplasty (TKA) [8, 9]. Nevertheless, consensus holds that effective preoperative rehabilitation hinges upon appropriate training duration, sufficient intensity, and rational exercise modalities [9–13]. Domínguez-Navarro et al. [14] and Gränicher et al. [15] established that 4 weeks of intensive preoperative training suffices to significantly improve muscle strength, balance, and function preoperatively, defining this period as the minimal effective dose. High-intensity, distributed-load regimens maximize neuromuscular adaptation within this 4-week window, expanding preoperative functional capacity [12, 13, 16]. Consequently, this study adopted a moderate 4-week duration for the prehabilitation intervention, aiming to optimally balance the requirement for sufficient physiological adaptation with pragmatic considerations of patient compliance and efficient healthcare resource allocation.

With societal aging driving a sustained increase in TKA procedures globally, there is growing interest in identifying efficient, accessible prehabilitation approaches. However, robust evidence specifically supporting the efficacy of a structured, moderate-duration (4-week), home-executable prehabilitation program within real-world clinical pathways is limited. This study therefore aimed to evaluate whether a structured 4-week home-based prehabilitation program would accelerate early postoperative recovery in patients undergoing TKA.

## Methods

### Ethical approval

This study was conducted as a retrospective cohort analysis in accordance with the ethical principles outlined in the Declaration of Helsinki. The decision to undergo preoperative rehabilitation was based on patient preference to mirror real-world clinical practice. Written informed consent was obtained from all participants prior to their inclusion in the study. The study was approved with the approval no. of ethic committee: K2021-12-060.

### Patient selection

**Fig. 1:**
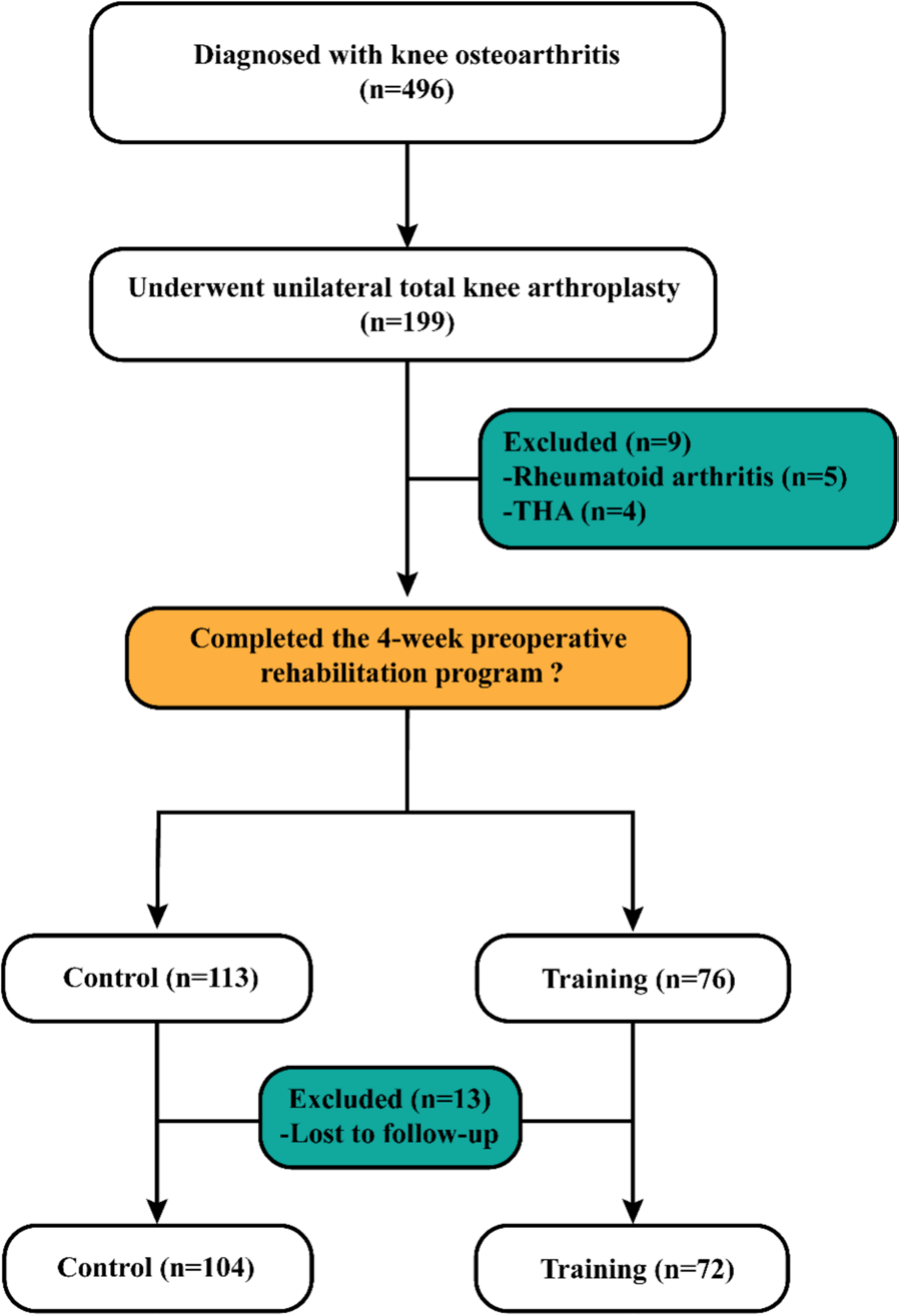
Inclusion and exclusion of patients. THA, total hip arthroplasty

This study included patients undergoing primary unilateral TKA for knee osteoarthritis at our center from January 2022 to January 2024 (Fig. 1). Considering that the preoperative rehabilitation training period in previous studies was mostly 4–8 weeks, patients who completed a 4-week home-based prehabilitation program (training group) and those who did not (control group) were selected as the subjects of this study. Inclusion criteria included: (1) preoperative diagnosis of unilateral knee osteoarthritis, Kellgren–Lawrence (K-L) III–IV; (2) receiving unilateral total knee arthroplasty; (3) clear consciousness, normal communication skills, and no cognition and communication barriers (to ensure their capability to understand and adhere to the home-based training regimen); and (4) completion of the 4-week preoperative rehabilitation program. Exclusion criteria were as follows: (1) patients with knee arthritis caused by other diseases, such as rheumatoid arthritis; (2) patients who have undergone other joint arthroplasty surgeries; (3) patients with bilateral total knee arthroplasty surgery; and (4) patients suffering from multiorgan failure and other conditions that impact daily activities.

### Components of preoperative rehabilitation training

Preoperatively, every patient underwent standardized health education, anti-osteoporosis treatment, and on-demand analgesia. All patients received standardized exercise instruction from the same trained physiotherapist team to ensure consistency. A “home-based exercise program” that comprised: (1) ankle-pumping: actively dorsiflex and plantarflex the ankle to maximal angles, maintain for 5 s, release for 5 s, and complete 20 reps per set; (2) isometric quadriceps setting: lying supine, lift the heel to tighten the thigh muscles with the patella stabilized, sustain contraction for 5 s, relax for 5 s, and perform 20 reps per set; (3) straight-leg lifts: with the knee fully extended, elevate the leg 20 cm off the bed, maintain for 10 s, return to start, and complete 20 reps per set with 5 s rest between lifts; (4) seated active range-of-motion (ROM) knee exercise: maximally flex the knee and hold for 5 s, then fully extend and hold for 5 s before returning to neutral, and repeat 20 times per set; (5) passive ROM knee exercise: while seated, gently push the operated leg into further flexion with the contralateral leg, maintain for 5 s, then relax to the initial position, and perform 20 repetitions per set; (6) standing hamstring training: with the knee fully extended, lift the lower limb backward to the maximal range, sustain for 20 s, then return to starting position, and complete 20 repetitions per set, resting 5 s between each; and (7) stationary marching: sustain upright balance and promptly address any gait deviations, and continue for 2 min each time. Prior to total knee arthroplasty, patients trained 5 days per week, performing the aforementioned exercises in three sets daily. A physiotherapist distributed training manuals to all patients and conducted weekly telephone check-ins to monitor progress, address questions, and verbally confirm completion of the prescribed exercises. After total knee arthroplasty, all participants followed the same postoperative rehabilitation protocol. Briefly, it typically begins in the hospital with pain control, basic mobility, and range-of-motion exercises, then advances over 3–6 months to outpatient or home-based strengthening, balance, and functional training aimed at restoring independence and activity.

### Surgical technique

All surgical procedures were performed by a single, consistent surgical team under general anesthesia via a sub-vastus medialis approach, utilizing the cruciate retaining (CR) prosthesis (Johnson, USA). In brief, removal of arthritic synovium and the anterior cruciate ligament were undertaken, with the posterior cruciate ligament deliberately retained. Osteophytes on the medial femoral condyle and medial tibial plateau were meticulously removed. Patellar osteophytes were excised and the articular surface was reshaped (patelloplasty) in all cases. The alignment strategy, either kinematic alignment (KA) or mechanical alignment (MA), was determined on an individualized basis according to the preoperative evaluation. Postoperatively, all patients were managed according to a standard perioperative management protocol.

### Measures

All participants completed a baseline practice session and were then evaluated at six distinct time points. At every visit, the following measures were collected: 10-cm visual analog scale (VAS) under resting (resting VAS) or active conditions (active VAS) [12], active knee ROM [17], Knee Society Score (KSS), timed up-and-go (TUG) test [18], stair‑climbing test [19], and the Western Ontario and McMaster Universities Arthritis Index (WOMAC) [20]. In the aforementioned assessment metrics, WOMAC served as the primary outcome measure. Each outcome was evaluated at baseline (4 weeks pre-TKA) and repeated on the day prior to surgery. Postoperatively, VAS was measured at 1 day, 1 week, 1 month, and 3 months, whereas the other indices were evaluated at 1, 3, 6, and 12 months. A single examiner, blinded to the training allocation, performed all evaluations to reduce the risk of bias.

### Sample size calculation

On the basis of the effect of prehabilitation on WOMAC scores in previous studies (with a standard deviation of 11) [21], and assuming a true difference in means between the intervention and control groups of at least 10, a sample size of 20 patients per group is sufficient to reject the null hypothesis, with a statistical power of 80% and a significance level of *p* < 0.05.

### Data analyses

Statistical analyses were conducted with SPSS version 22.0 (IBM Corp., Armonk, NY, USA). Normality was assessed using the Shapiro–Wilk test along with visual inspection of Q–Q plots for remaining variables; continuous data conforming to a normal distribution were presented as mean ± standard deviation (SD) and analyzed with independent *t*-tests. For non-normally distributed variables, medians with interquartile ranges [median (lower quartile, upper quartile)] were reported and group differences assessed with the Mann–Whitney *U* test. Categorical variables were compared using the chi-squared test or Fisher’s exact test as appropriate. Variations in outcome measures were analyzed via a repeated-measures linear mixed model that included group, time, and their interaction as fixed effects. The model was adjusted for the following prespecified covariates: age, sex, body mass index (BMI), age-adjusted Charlson Comorbidity Index (aCCI), Kellgren–Lawrence grade, surgical side, and alignment strategy (kinematic versus mechanical). Statistical significance was set at *P* < 0.05.

## Results

### Patient demographic characteristics

A total of 176 patients were included and completed over 1 year of follow-up: 104 in the control group and 72 in the training group. No significant differences were observed between the two groups regarding baseline demographics, including age, sex, body mass index (BMI), age-adjusted Charlson Comorbidity Index (aCCI), surgical side, or Kellgren–Lawrence (K-L) radiographic grade and alignment strategy (Table 1).

**Table 1:** Analysis of the demographics and preoperative characteristics of the two groups

Characteristic	Control (*n* = 104)	Training (*n* = 72)	*P*-value	Difference (95% CI)
Gender, *n* (%)			0.360^a^	OR = 0.71 (0.34, 1.50)
Male	20 (19.2%)	18 (25.0%)		
Female	84 (80.8%)	54 (75.0%)		
Age, years (mean ± SD)	69.6 ± 7.5	69.0 ± 6.8	0.595^b^	MD = 0.60 (−1.60, 2.80)
BMI, kg/m^2^ (mean ± SD)	25.2 ± 3.5	25.9 ± 3.3	0.203^b^	MD = −0.66 (−1.68, 0.36)
aCCI scores (median (LQR))	2.00 (1, 4)	2.00 (1, 4)	0.716^c^	MedD = 0.00 (−0.50, 0.50)
Operated side, *n* (%)			0.944^a^	OR = 1.02 (0.56, 1.86)
Left	50 (48.1%)	35 (48.6%)		
Right	54 (51.9%)	37 (51.4%)		
Stages of KOA, *n* (%)			0.762^a^	OR = 0.91 (0.47, 1.74)
K-L grade III	31 (29.8%)	23 (31.9%)		
K-L grade IV	73 (70.2%)	49 (68.1%)		
Alignment strategy			0.967^a^	OR = 1.01 (0.55, 1.85)
KA, *n* (%)	48 (46.2%)	33 (45.8%)		
MA, *n* (%)	56 (53.8%)	39 (54.2%)		

*SD* standard deviation, *BMI* body mass index, *IQR* interquartile range, *aCCI* age-adjusted Charlson Comorbidity Index, *KOA* knee osteoarthritis, *K-L grade* Kellgren–Lawrence grade, *KA* kinematic alignment, *MA* mechanical alignment, *OR* odds ratio, *MD* mean difference, *MedD* median difference

^a^Chi-squared test

^b^Independent *t*-test

^c^Mann-Whitney *U* test

### Perioperative outcomes

No significant between-group differences were observed in operative duration, intraoperative blood loss, or total inpatient expenses (Table 2). Notably, patients of training group achieved earlier first ambulation postoperatively (median difference: −3 h; *P* = 0.003; Table 2) and exhibited a significantly reduced length of hospital stay compared with patients of the control group (median difference: −3 days; *P* = 0.002; Table 2).

**Table 2:** Hospitalization related information of the two groups

	Control (*n* = 104)	Training (*n* = 72)	*P*-value	Difference (95% CI)
Operation time, min [median (IQR)]	130 (62, 240)	130 (83, 270)	0.587^c^	MedD = −2.00 (−10.00, 6.00)
Blood loss, ml [median (IQR)]	150 (10, 1000)	200 (20, 600)	0.066^c^	MedD = −0.00 (−50.00, 0.00)
Time to out-of-bed, hours [median (IQR)]	12 (1, 22)	9 (1, 20)	0.003^c^	MedD = 2.00 (1.00, 4.00)
Length of stay, days [median (IQR)]	10 (2, 25)	7 (2, 21)	0.002^c^	MedD = 2.00 (1.00, 3.00)
Hospital costs, 10^4^ CNY [median (IQR)]	4.9 (2.0, 8.2)	4.1 (1.7, 7.4)	0.124^c^	MedD = 2.48 (−6.53, 8.62)

*IQR* interquartile range, *MedD* median difference

^c^ Mann–Whitney *U* test

### Pain assessment

**Fig. 2:**
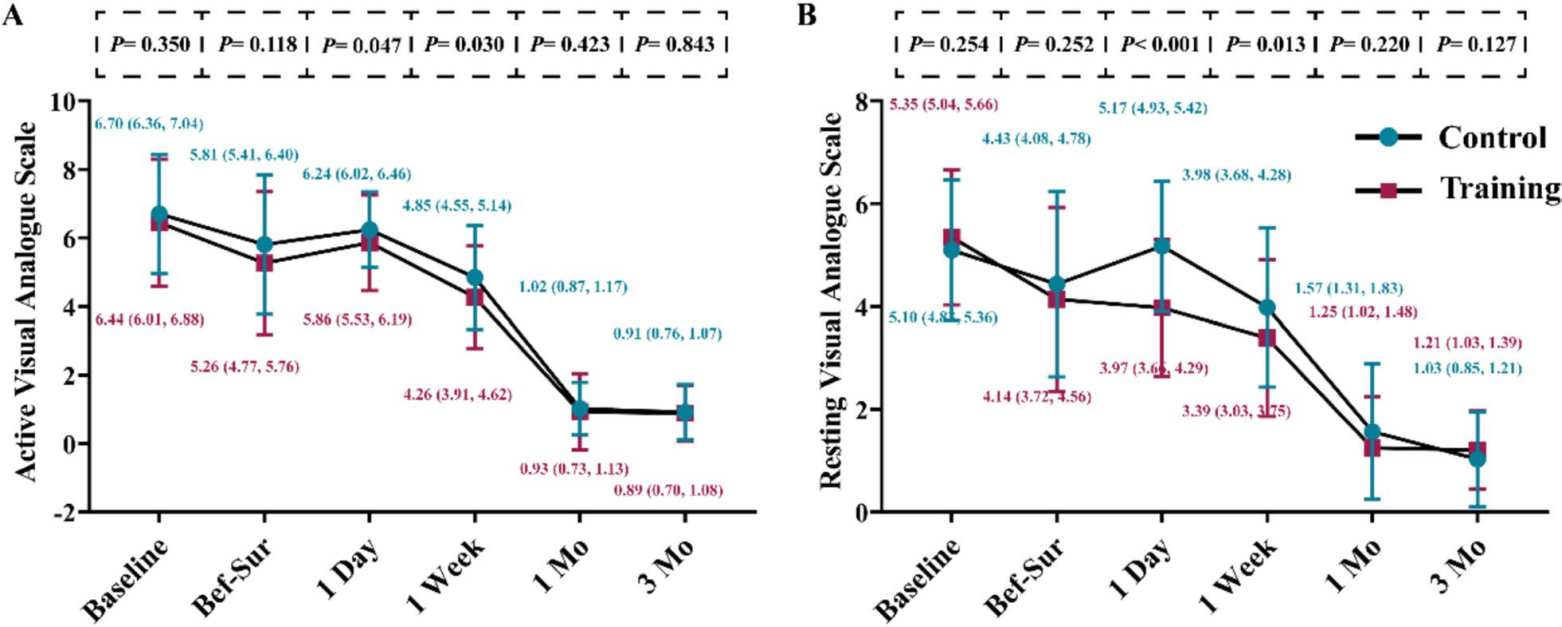
Visual analog scale (VAS) scores over the 12-month follow-up period. Postoperative knee pain was assessed using the VAS, with separate assessments for active VAS (**A**) and resting VAS (**B**). Data are presented as point estimates with 95% confidence intervals in parentheses. Statistical analysis: Mann–Whitney *U* test. *Bef-Sur* before surgery, *Mo* month

After adjusting for covariates (age, sex, BMI, aCCI, K-L grade, joint involvement, and alignment strategy) in a repeated-measures linear mixed model, the training group demonstrated significantly lower resting VAS and active VAS scores during the first postoperative week (*P* < 0.05 for group-by-time interaction). Post hoc between-group comparisons at individual time points confirmed significantly lower pain scores in the training group at 1 day and 1 week postoperatively (*P* < 0.05). No significant between-group differences in pain scores were observed from 1 month onward. Detailed scores and statistical comparisons are presented in Fig. 2 and Supplementary Table S1.

### Functional and physical measures

**Fig. 3:**
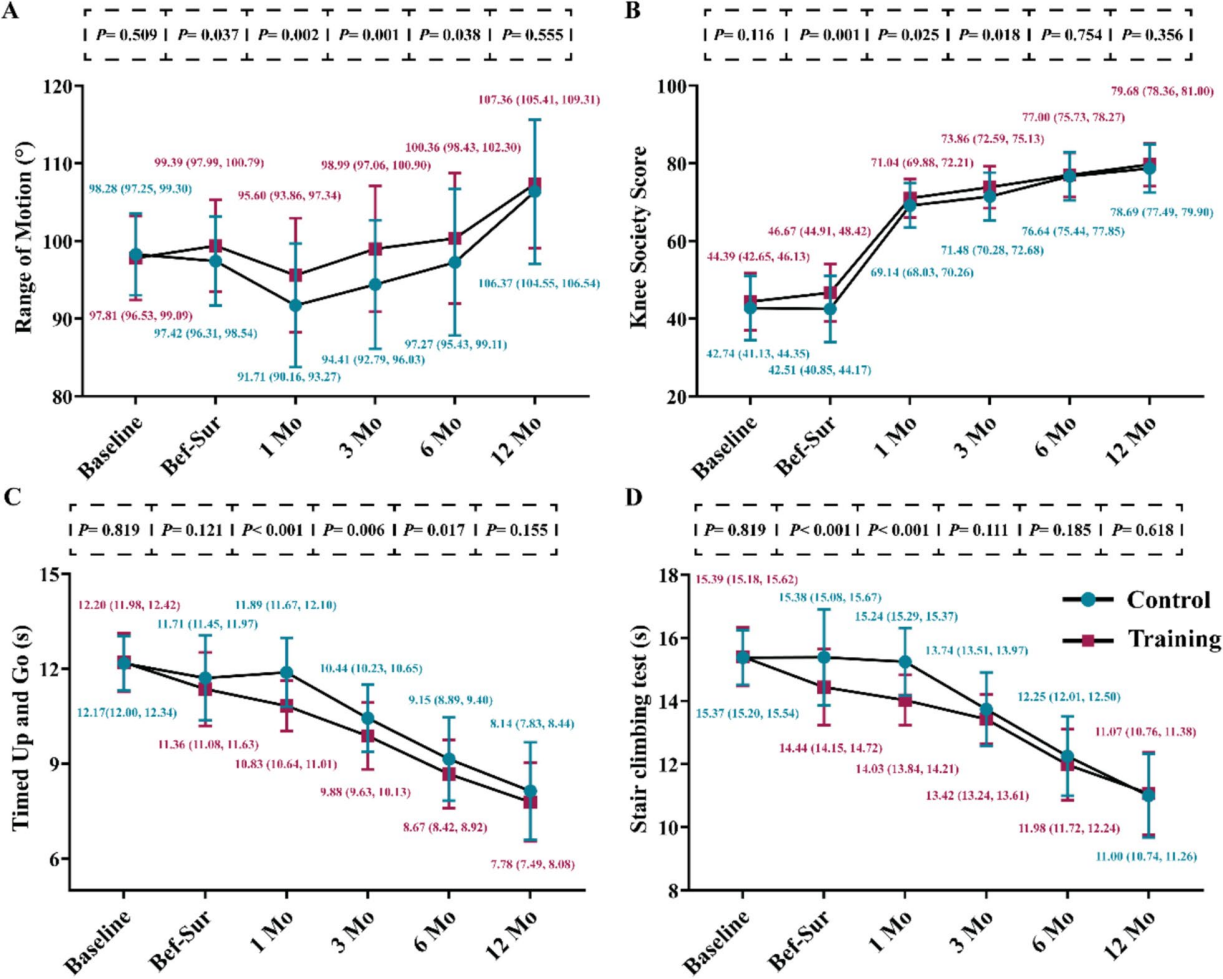
Scores for all physical measures over the 12-month follow-up period. Postoperative physical function was assessed using a battery of tests, including active knee range of motion (**A**), the Knee Society Score (**B**), the timed up-and-go test (**C**), and a stair-climbing test (**D**). Data are presented as point estimates with 95% confidence intervals in parentheses. Statistical analysis: Mann–Whitney *U* test. *Bef-Sur* before surgery, *Mo* month

4.1. Preoperative status: following the 4-week prehabilitation program, the training group showed significant improvements in knee ROM, KSS, and stair-climbing test compared with the control group (*P* < 0.05).
4.2. Postoperative recovery: from 1 to 3 months postoperatively, the training group exhibited superior recovery compared with the control group across multiple functional metrics. They had significantly greater active knee ROM, higher KSS, and faster TUG test times at both 1 and 3 months (*P* < 0.05 for all). The advantage in the stair-climbing test was significant at 1 month (*P* < 0.001). These findings remained significant after adjustment for covariates in the linear mixed model (group-by-time interaction *P* < 0.05). The benefits in ROM and TUG performance persisted and remained statistically significant at the 6-month follow-up (*P* < 0.05). By 12 months postoperatively, all functional outcomes, including ROM, KSS, TUG, and the stair test, had converged, with no significant differences between the groups (all *P* > 0.05). Complete data for all time points are available in Fig. 3 and Supplementary Table S2.

### Patient-reported outcomes

**Fig. 4:**
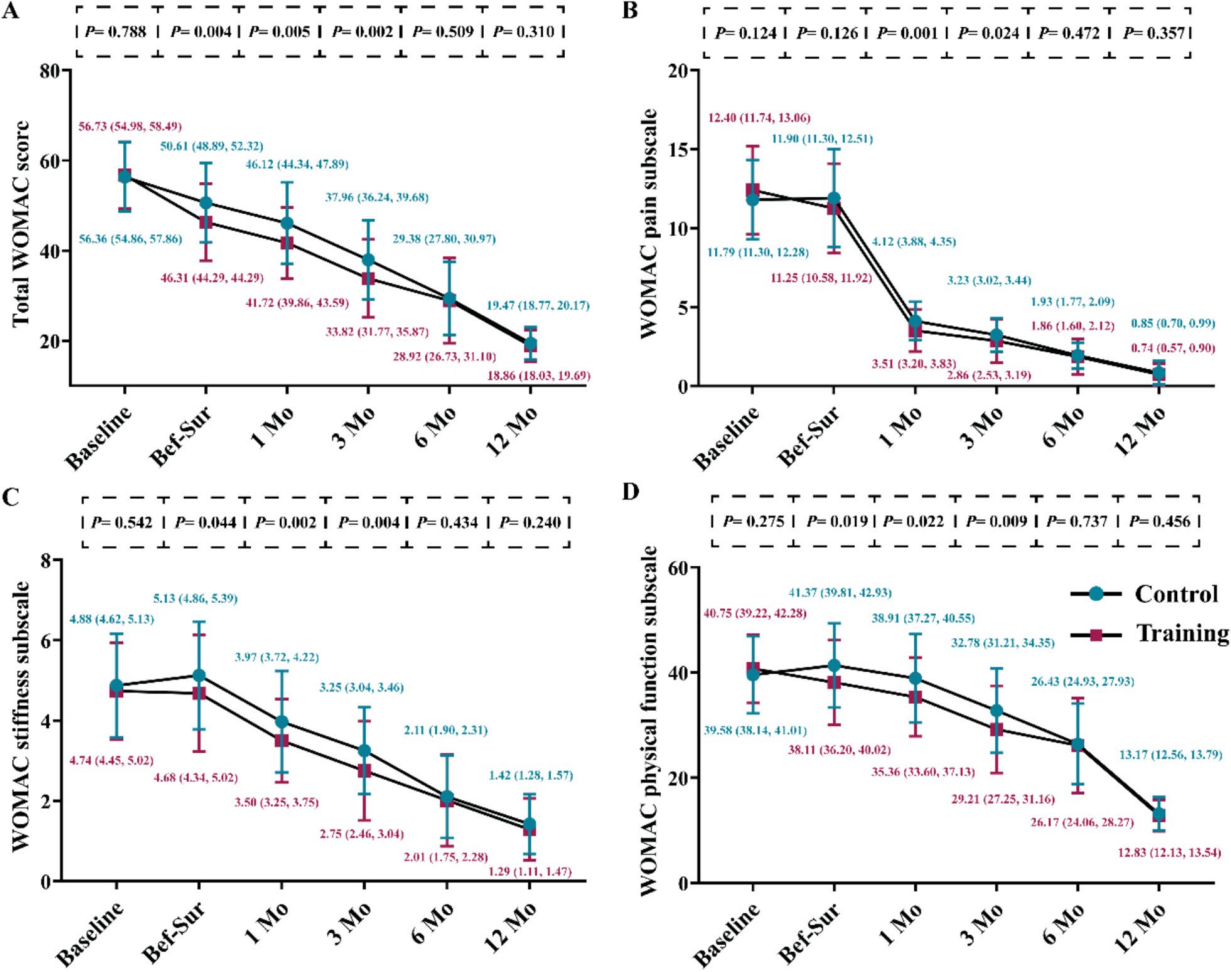
Western Ontario and McMaster Universities Osteoarthritis Index (WOMAC) Scores over the 12-month follow-up period. Postoperative recovery was evaluated using the WOMAC, which provides a total score (**A**) and subscores for pain (**B**), stiffness (**C**), and physical function (**D**). Higher scores on the WOMAC scale indicate greater pain and stiffness severity, as well as poorer physical function. Data are presented as point estimates with 95% confidence intervals in parentheses. Statistical analysis: Mann–Whitney *U* test. *Bef-Sur* before surgery, *Mo* month

To further assess the influence of the preoperative rehabilitation program on early postoperative recovery following TKA, we performed statistical analysis on data derived from the WOMAC questionnaire (Fig. 4 and Supplementary Table S3). After prehabilitation, the training group reported significantly better preoperative WOMAC scores for stiffness and function compared with the control group (*P* < 0.05). Postoperatively, the training group maintained a significant advantage in total WOMAC score and its subscales (pain, stiffness, and function) at both 1 and 3 months (*P* < 0.05). This superior recovery trajectory up to 3 months was confirmed by the repeated-measures model (*P* < 0.05 for group-by-time interaction). No significant between-group differences in WOMAC scores were observed at 6 or 12 months postoperatively.

## Discussion

This retrospective cohort study observed an association between a 4-week home-based prehabilitation program and accelerated early functional recovery. The key findings, including earlier ambulation, shorter hospital stay, and superior patient-reported function as measured by the WOMAC score at the 3-month follow-up, support further investigation into integrating such time-efficient programs into clinical pathways.

The results align with the evolving evidence on prehabilitation in orthopedic surgery. The reduction in time to first ambulation and length of hospital stay (by a median of 3 days) is clinically meaningful and comparable to benefits reported with more intensive or longer-duration programs [13, 22]. Although the length of stay and its reduction are directly influenced by local perioperative protocols and healthcare system practices, the relative association between prehabilitation and accelerated recovery may still hold. Furthermore, the marked reduction in pain scores during the critical first postoperative week and the consistent functional advantages (ROM, KSS, TUG) observed up to 3 months corroborate findings from recent meta-analyses and RCTs [13, 14, 22–24]. This is consistent with the theoretical framework suggesting that preoperative conditioning may facilitate early postoperative neuromuscular activation and thereby contribute to accelerated functional recovery [25, 26]. The early superiority in WOMAC scores, our primary outcome, further strengthens the argument for prehabilitation’s role in improving the early patient experience after TKA.

Notably, the convergence of all outcome measures between groups by 12 months postoperatively is a critical observation. This pattern, also reported in long-term follow-up studies [13, 27], suggests that the principal value of prehabilitation lies in accelerating the recovery trajectory rather than altering the final, long-term functional plateau. Standardized postoperative rehabilitation and the natural healing process appear to allow control patients to “catch up” over time. Therefore, the clinical and economic justification for prehabilitation may hinge predominantly on its ability to reduce early morbidity and resource use.

This study highlights several clinically relevant strengths. First, the home-based nature of the program enhances accessibility, reduces patient burden, and improves scalability within real-world healthcare systems. The exercises prescribed are simple, equipment-free, and easy to teach, promoting adherence. Second, focusing on a 4-week duration represents a pragmatic compromise. It provides sufficient time for physiological adaptation while being logistically feasible and likely to maintain patient compliance better than longer protocols, which often suffer from attrition [28]. Third, the enrolled cohort consisted of patients with advanced radiographic osteoarthritis (K-L grades III–IV) and a mean age of 66 years. This population, with lower baseline function, may derive greater absolute benefit from prehabilitation by augmenting physiological reserve, supporting the “worse baseline, greater benefit” hypothesis often discussed in rehabilitation literature [13, 24]. The clinical relevance of the observed differences is supported by comparisons to established minimal clinically important difference (MCID) thresholds. The between-group difference in WOMAC total score at 3 months (approximately 4–5 points) approaches the reported MCID range of 6–12 points for TKA patients. Furthermore, the difference in TUG test time at 3 months (approximately 1 s) meets or exceeds the commonly cited MCID of 1 s, suggesting a meaningful improvement in functional mobility [14, 23, 24].

Several limitations must be acknowledged. First, the retrospective cohort design with allocation based on patient preference introduces the potential for selection bias. Although baseline characteristics were balanced and analyses were adjusted for key covariates, unmeasured confounding factors (e.g., intrinsic motivation, pre-existing activity levels, psychosocial factors) could influence outcomes. Second, the lack of a uniform surgical alignment technique (KA versus MA) across the cohort introduces a source of procedural heterogeneity. While the choice was individualized on the basis of anatomical criteria, it constitutes a methodological variability. Third, this study evaluated multiple secondary outcome measures. While the use of a mixed model for each outcome appropriately handles comparisons across time within that measure, the assessment of several different outcomes without adjustment for multiple comparisons increases the risk of type I error. Consequently, the results for secondary outcomes should be regarded as exploratory and generating hypotheses for future research. Fourth, owing to its retrospective design, this study could not accurately ascertain actual patient adherence within the target population. Moreover, although completion was monitored via training manuals and weekly telephone checks, these assessments relied solely upon self-report without objective validation (e.g., through wearable sensors or exercise logs). Future investigations should optimize study design and incorporate objective adherence monitoring to establish clearer dose–response relationships. Finally, the unrecorded variability in postoperative rehabilitation intensity as a potential unmeasured confounder.

On the basis of these limitations, future research should: (1) conduct a multicenter, randomized controlled trial to robustly establish the cost-effectiveness of this 4-week protocol; (2) employ wearable sensor technology to objectively quantify early postoperative gait parameters, overcoming the ceiling effects of traditional clinical scales; and (3) investigate strategies for seamless integration of preoperative training with structured early postoperative telerehabilitation to potentially extend the window of accelerated recovery.

## Conclusions

In conclusion, a 4-week home-based prehabilitation program is a feasible and effective strategy to enhance early recovery after TKA. It facilitates faster mobilization, shortens hospitalization, and improves pain and function within the early stage, supporting its inclusion in standardized ERAS protocols for patients undergoing knee arthroplasty.

## Supplementary Information

Supplementary material 1.

Supplementary material 2.

## Data Availability

The datasets used and/or analyzed during the current study are available from the corresponding author on reasonable request.

## Abbreviations

OA: Knee osteoarthritis
TKA: Total knee arthroplasty
ERAS: Enhanced recovery after surgery
K-L: Kellgren–Lawrence
ROM: Range-of-motion
CR: Cruciate retaining
VAS: Visual analog scale
KSS: Knee Society Score
TUG: Timed up-and-go
WOMAC: Western Ontario and McMaster Universities Arthritis Index
BMI: Body mass index
aCCI: Age-adjusted Charlson Comorbidity Index
