# Supplementary material for: Significance of a 4-week home-based prehabilitation program in accelerating 3-month recovery post total knee arthroplasty: a retrospective cohort study
Source: Knee Surg Relat Res. 2026 Jul 1;38:21. doi: 10.1186/s43019-026-00315-7 (PMC13321678; doi:10.1186/s43019-026-00315-7)
Supplement: Supplementary file 1 — Supplementary material 1. [file 43019_2026_315_MOESM1_ESM.docx]

**Supplementary materials**

**Significance of a 4-Week Home-Based Prehabilitation Program in Accelerating 3-Month Recovery Post Total Knee Arthroplasty: A Retrospective Cohort Study**

Table S1 Evaluation of Visual Analog Scale (VAS) scores between groups postoperatively.

| Characteristic | Control (n=104) | Training (n=72) | *P* value | *P* value |
| --- | --- | --- | --- | --- |
| Resting VAS | mean (95 % CI) | mean (95 % CI) |  |  |
| Baseline | 5.10 (4.83, 5.36) | 5.35 (5.04, 5.66) | 0.254 ^c^ | / |
| Before surgery | 4.43 (4.08, 4.78) | 4.14 (3.72, 4.56) | 0.252 ^c^ | / |
| 1 day after surgery | 5.17 (4.93, 5.42) | 3.97 (3.66, 4.29) | <0.001 ^c^ | / |
| 1 week after surgery | 3.98 (3.68, 4.28) | 3.39 (3.03, 3.75) | 0.013 ^c^ | 0.019 ^d^ |
| 1 month after surgery | 1.57 (1.31, 1.83) | 1.25 (1.02, 1.48) | 0.220 ^c^ | 0.106^d^ |
| 3 months after surgery | 1.03 (0.85, 1.21) | 1.21 (1.03, 1.39) | 0.127 ^c^ | 0.149 ^d^ |
| Active VAS |  |  |  |  |
| Baseline | 6.70 (6.36, 7.04) | 6.44 (6.01, 6.88) | 0.350 ^c^ | / |
| Before surgery | 5.81 (5.41, 6.40) | 5.26 (4.77, 5.76) | 0.118 ^c^ | / |
| 1 day after surgery | 6.24 (6.02, 6.46) | 5.86 (5.53, 6.19) | 0.047 ^c^ | / |
| 1 week after surgery | 4.85 (4.55, 5.14) | 4.26 (3.91, 4.62) | 0.030 ^c^ | 0.013 ^d^ |
| 1 month after surgery | 1.02 (0.87, 1.17) | 0.93 (0.73, 1.13) | 0.423 ^c^ | 0.536^d^ |
| 3 months after surgery | 0.91 (0.76, 1.07) | 0.89 (0.70, 1.08) | 0.843 ^c^ | 0.939 ^d^ |

^c^ Mann-Whitney U test; ^d^ Repeated measures linear mixed models.

Table S2 Scores for all physical measures postoperatively.

| Characteristic | Control (n=104) | Training (n=72) | *P* value | *P* value |
| --- | --- | --- | --- | --- |
| ROM, ° | mean (95 % CI) | mean (95 % CI) |  |  |
| Baseline | 98.28 (97.25, 99.30) | 97.81 (96.53, 99.09) | 0.509 ^c^ | / |
| Before surgery | 97.42 (96.31, 98.54) | 99.39 (97.99, 100.79) | 0.037 ^c^ | / |
| 1 month after surgery | 91.71 (90.16, 93.27) | 95.60 (93.86, 97.34) | 0.002 ^c^ | / |
| 3 months after surgery | 94.41 (92.79, 96.03) | 98.99 (97.06, 100.90) | 0.001 ^c^ | <0.001^d^ |
| 6 months after surgery | 97.27 (95.43, 99.11) | 100.36 (98.43, 102.30) | 0.038 ^c^ | 0.027 ^d^ |
| 12 months after surgery | 106.37 (104.55, 106.54) | 107.36 (105.41, 109.31) | 0.555 ^c^ | 0.480 ^d^ |
| KSS |  |  |  |  |
| Baseline | 42.74 (41.13, 44.35) | 44.39 (42.65, 46.13) | 0.166 ^c^ | / |
| Before surgery | 42.51 (40.85, 44.17) | 46.67 (44.91, 48.42) | 0.001 ^c^ | / |
| 1 month after surgery | 69.14 (68.03, 70.26) | 71.04 (69.88, 72.21) | 0.025 ^c^ | / |
| 3 months after surgery | 71.48 (70.28, 72.68) | 73.86 (72.59, 75.13) | 0.018 ^c^ | 0.012 ^d^ |
| 6 months after surgery | 76.64 (75.44, 77.85) | 77.00 (75.73, 78.27) | 0.754 ^c^ | 0.709 ^d^ |
| 12 months after surgery | 78.69 (77.49, 79.90) | 79.68 (78.36, 81.00) | 0.356 ^c^ | 0.295 ^d^ |
| TUG test, s |  |  |  |  |
| Baseline | 12.17(12.00, 12.34) | 12.20 (11.98, 12.42) | 0.819 ^c^ | / |
| Before surgery | 11.71 (11.45, 11.97) | 11.36 (11.08, 11.63) | 0.121 ^c^ | / |
| 1 month after surgery | 11.89 (11.67, 12.10) | 10.83 (10.64, 11.01) | <0.001 ^c^ | / |
| 3 months after surgery | 10.44 (10.23, 10.65) | 9.88 (9.63, 10.13) | 0.006 ^c^ | <0.001^d^ |
| 6 months after surgery | 9.15 (8.89, 9.40) | 8.67 (8.42, 8.92) | 0.017 ^c^ | 0.009 ^d^ |
| 12 months after surgery | 8.14 (7.83, 8.44) | 7.78 (7.49, 8.08) | 0.155 ^c^ | 0.096 ^d^ |
| Stair-climbing test, s |  |  |  |  |
| Baseline | 15.37 (15.20, 15.54) | 15.39 (15.18, 15.62) | 0.819 ^c^ | / |
| Before surgery | 15.38 (15.08, 15.67) | 14.44 (14.15, 14.72) | <0.001 ^c^ | / |
| 1 month after surgery | 15.24 (15.29, 15.37) | 14.03 (13.84, 14.21) | <0.001 ^c^ | / |
| 3 months after surgery | 13.74 (13.51, 13.97) | 13.42 (13.24, 13.61) | 0.111 ^c^ | 0.034 ^d^ |
| 6 months after surgery | 12.25 (12.01, 12.50) | 11.98 (11.72, 12.24) | 0. 185 ^c^ | 0.125 ^d^ |
| 12 months after surgery | 11.00 (10.74, 11.26) | 11.07 (10.76, 11.38) | 0.618 ^c^ | 0.785 ^d^ |

TUG test, Timed Up and Go test.

^c^ Mann-Whitney U test; ^d^ Repeated measures linear mixed models.

Table S3 Evaluation of clinical outcomes between groups postoperatively.

| Characteristic | Control (n=104) | Training (n=72) | *P* value | *P* value |
| --- | --- | --- | --- | --- |
| WOMAC | mean (95 % CI) | mean (95 % CI) |  |  |
| Baseline | 56.36 (54.86, 57.86) | 56.73 (54.98, 58.49) | 0.788 ^c^ | / |
| Before surgery | 50.61 (48.89, 52.32) | 46.31 (44.29, 44.29) | 0.004 ^c^ | / |
| 1 month after surgery | 46.12 (44.34, 47.89) | 41.72 (39.86, 43.59) | 0.005 ^c^ | / |
| 3 months after surgery | 37.96 (36.24, 39.68) | 33.82 (31.77, 35.87) | 0.002 ^c^ | 0.001 ^d^ |
| 6 months after surgery | 29.38 (27.80, 30.97) | 28.92 (26.73, 31.10) | 0.509 ^c^ | 0.613 ^d^ |
| 12 months after surgery | 19.47 (18.77, 20.17) | 18.86 (18.03, 19.69) | 0.310 ^c^ | 0.222 ^d^ |
| WOMACpain |  |  |  |  |
| Baseline | 11.79 (11.30, 12.28) | 12.40 (11.74, 13.06) | 0.124 ^c^ | / |
| Before surgery | 11.90 (11.30, 12.51) | 11.25 (10.58, 11.92) | 0.126 ^c^ | / |
| 1 month after surgery | 4.12 (3.88, 4.35) | 3.51 (3.20, 3.83) | 0.001 ^c^ | / |
| 3 months after surgery | 3.23 (3.02, 3.44) | 2.86 (2.53, 3.19) | 0.024 ^c^ | 0.044 ^d^ |
| 6 months after surgery | 1.93 (1.77, 2.09) | 1.86 (1.60, 2.12) | 0.472 ^c^ | 0.614 ^d^ |
| 12 months after surgery | 0.85 (0.70, 0.99) | 0.74 (0.57, 0.90) | 0.357 ^c^ | 0.290 ^d^ |
| WOMACstiffness |  |  |  |  |
| Baseline | 4.88 (4.62, 5.13) | 4.74 (4.45, 5.02) | 0.542 ^c^ | / |
| Before surgery | 5.13 (4.86, 5.39) | 4.68 (4.34, 5.02) | 0.044 ^c^ | / |
| 1 month after surgery | 3.97 (3.72, 4.22) | 3.50 (3.25, 3.75) | 0.002 ^c^ | / |
| 3 months after surgery | 3.25 (3.04, 3.46) | 2.75 (2.46, 3.04) | 0.004 ^c^ | 0.003 ^d^ |
| 6 months after surgery | 2.11 (1.90, 2.31) | 2.01 (1.75, 2.28) | 0.434 ^c^ | 0.513 ^d^ |
| 12 months after surgery | 1.42 (1.28, 1.57) | 1.29 (1.11, 1.47) | 0.240 ^c^ | 0.218 ^d^ |
| WOMACfunctional |  |  |  |  |
| Baseline | 39.58 (38.14, 41.01) | 40.75 (39.22, 42.28) | 0.275 ^c^ | / |
| Before surgery | 41.37 (39.81, 42.93) | 38.11 (36.20, 40.02) | 0.019 ^c^ | / |
| 1 month after surgery | 38.91 (37.27, 40.55) | 35.36 (33.60, 37.13) | 0.022 ^c^ | / |
| 3 months after surgery | 32.78 (31.21, 34.35) | 29.21 (27.25, 31.16) | 0.009 ^c^ | 0.002 ^d^ |
| 6 months after surgery | 26.43 (24.93, 27.93) | 26.17 (24.06, 28.27) | 0.737 ^c^ | 0.733 ^d^ |
| 12 months after surgery | 13.17 (12.56, 13.79) | 12.83 (12.13, 13.54) | 0.456 ^c^ | 0.422 ^d^ |

WOMAC, Western Ontario and McMaster Universities Osteoarthritis Index.

^c^ Mann-Whitney U test; ^d^ Repeated measures linear mixed models.
