# Supplementary material for: Significance of a 4-week home-based prehabilitation program in accelerating 3-month recovery post total knee arthroplasty: a retrospective cohort study
Source: Knee Surg Relat Res. 2026 Jul 1;38:21. doi: 10.1186/s43019-026-00315-7 (PMC13321678; doi:10.1186/s43019-026-00315-7)
Supplement: Supplementary file 2 — Supplementary material 2. [file 43019_2026_315_MOESM2_ESM.docx]

**Home Exercise Program Weekly Log (Week: 1- 4 )**

**Name:** ____________________

**Instructions:** Please fill in the dates of your 5 training days below. After completing each set of exercises, mark one checkbox. Your goal is to check all 3 boxes for each exercise on your training days.

| Exercise | Key Instruction | **Day 1:**  **/** | **Day 2:**  **/** | **Day 3:**  **/** | **Day 4:**  **/** | **Day 5:**  **/** |
| --- | --- | --- | --- | --- | --- | --- |
| **1. Ankle Pumping** | 20 reps/set | ☐ ☐ ☐ | ☐ ☐ ☐ | ☐ ☐ ☐ | ☐ ☐ ☐ | ☐ ☐ ☐ |
| **2. Quadriceps Setting** | 20 reps/set | ☐ ☐ ☐ | ☐ ☐ ☐ | ☐ ☐ ☐ | ☐ ☐ ☐ | ☐ ☐ ☐ |
| **3. Straight Leg Raises** | 20 reps/set | ☐ ☐ ☐ | ☐ ☐ ☐ | ☐ ☐ ☐ | ☐ ☐ ☐ | ☐ ☐ ☐ |
| **4. Seated Active ROM Knee** | 20 reps/set | ☐ ☐ ☐ | ☐ ☐ ☐ | ☐ ☐ ☐ | ☐ ☐ ☐ | ☐ ☐ ☐ |
| **5. Seated Passive ROM Knee** | 20 reps/set | ☐ ☐ ☐ | ☐ ☐ ☐ | ☐ ☐ ☐ | ☐ ☐ ☐ | ☐ ☐ ☐ |
| **6. Standing Hamstring** | 20 reps/set | ☐ ☐ ☐ | ☐ ☐ ☐ | ☐ ☐ ☐ | ☐ ☐ ☐ | ☐ ☐ ☐ |
| **7. Stationary Marching** | 2 min/set | ☐ ☐ ☐ | ☐ ☐ ☐ | ☐ ☐ ☐ | ☐ ☐ ☐ | ☐ ☐ ☐ |

**Notes / Comments (e.g., pain, swelling, fatigue):**

- **Day 1:** ______________________________________________________________
- **Day 2:**  _____________________________________________________________
- **Day 3:** ______________________________________________________________
- **Day 4:** ______________________________________________________________
- **Day 5:** ______________________________________________________________

**How to Use This Log:**

1. **Fill in Dates:** At the top of each "Day" column, write the specific date (e.g., Mon 4/10 or just 4/10).
2. **Check Boxes:** Each exercise requires **3 sets**. After you finish **Set 1**, check the first box. After **Set 2**, check the second box. After **Set 3**, check the third box.
3. **Notes Section:** Use this space to record any issues, such as increased pain, swelling, or if you felt particularly good that day.
